# Characterizing Ertapenem Neurotoxicity: A Systematic Review and Experience at a Tertiary Medical Center

**DOI:** 10.1093/ofid/ofae214

**Published:** 2024-04-16

**Authors:** Hayato Mitaka, Shinya Hasegawa, Kristine F Lan, Rupali Jain, Robert M Rakita, Paul S Pottinger

**Affiliations:** Division of Allergy and Infectious Diseases, Department of Medicine, University of Washington, Seattle, Washington, USA; Department of Internal Medicine, University of Iowa, Iowa City, Iowa, USA; Division of Allergy and Infectious Diseases, Department of Medicine, University of Washington, Seattle, Washington, USA; Division of Allergy and Infectious Diseases, Department of Medicine, University of Washington, Seattle, Washington, USA; School of Pharmacy, University of Washington, Seattle, Washington, USA; Division of Allergy and Infectious Diseases, Department of Medicine, University of Washington, Seattle, Washington, USA; Division of Allergy and Infectious Diseases, Department of Medicine, University of Washington, Seattle, Washington, USA

**Keywords:** β-lactams, carbapenems, ertapenem, neurotoxicity, systematic review

## Abstract

Ertapenem-induced neurotoxicity has not been well characterized and is potentially underreported. We conducted a systematic review of the literature and included 11 additional cases from the University of Washington Medicine health system. A total of 125 individual patient cases were included in the data analysis. The mean age was 72 years, and 62% and 42% of patients had renal dysfunction and preexisting central nervous system (CNS) conditions, respectively. Only 15% of patients received inappropriately high ertapenem dosing based on kidney function. Patients developed neurological signs and symptoms after a median of 4 days (interquartile range, 3–9 days). The most common clinical features were seizures (70%), altered level of consciousness or delirium (27%), and hallucinations (17%). An estimated incidence in our health system was 1 in 102 courses of ertapenem. Ertapenem neurotoxicity should be suspected when a patient with renal dysfunction or predisposing CNS conditions develops neurological signs and symptoms, especially within several days after initiating the antibiotic. This study underscores the need for a large prospective study to assess the true incidence and outcomes of ertapenem neurotoxicity.

Ertapenem is a carbapenem antibiotic that was approved in 2001 by the United States Food and Drug Administration (FDA) for the treatment of complicated intra-abdominal infections, complicated skin and skin structure infections, community-acquired pneumonia, complicated urinary tract infections including pyelonephritis, and acute pelvic and gynecological infections. Once-daily intravenous dosing provides broad-spectrum antimicrobial activity against anaerobes and many aerobic gram-positive and gram-negative organisms, including extended-spectrum β-lactamase (ESBL)– and AmpC β-lactamase–producing Enterobacterales [1–3]. Ertapenem is a convenient option for outpatient parenteral antibiotic therapy (OPAT) and is often continued for weeks [4, 5].

All β-lactam antibiotics have the potential to cause neurotoxicity by antagonizing central nervous system (CNS) γ-aminobutyric acid type A (GABA_A_) receptors, especially in patients with risk factors such as renal dysfunction, older age, and disruptions in the integrity of the blood-brain barrier [6]. Ertapenem is no exception: Cases of ertapenem neurotoxicity have been sporadically reported, such as seizures and encephalopathy. However, the incidence of ertapenem neurotoxicity relative to other β-lactam antibiotics remains unclear. Surprisingly, a recent pharmacovigilance study of the FDA adverse event reporting system demonstrated that among antibiotics evaluated in the study, including many other β-lactams commonly associated with neurotoxicity such as cefepime and imipenem, ertapenem had by far the highest delirium reporting rate [7]. This is particularly concerning because (1) little is known about the incidence, clinical presentations, and association with common risk factors of β-lactam neurotoxicity, and (2) ertapenem is commonly used for weeks in the outpatient setting, both of which could result in underdiagnoses and delays in discontinuation of the offending antibiotic.

In this systematic review, we synthesize the existing literature on ertapenem-induced neurotoxicity. We also provide clinicians with a framework to assist with early identification of neurotoxicity, focusing on risk factors, clinical manifestations, management, and outcomes. As a part of ongoing quality improvement activities, we also review all potential cases of ertapenem neurotoxicity in the University of Washington Medicine (UW Medicine) health system during a 2-year period.

## METHODS

This systematic review was conducted in accordance with the Preferred Reporting Items for Systematic Reviews and Meta-Analyses extension for scoping reviews (PRISMA-ScR) [8].

### Search Strategy and Eligibility Criteria

We searched Medline/PubMed and Embase for all peer-reviewed articles from inception through April 2023 using the following search terms: “ertapenem” AND (“Neurotoxicity” OR “Encephalopathy” OR “Seizures” OR “status epilepticus” OR “Convulsions” OR “Delirium” OR “Psychosis” OR “Hallucinations” OR “altered mental status” OR coma OR stupor OR obtundation). We also performed an additional manual search of references of initially identified articles, to maximize the completeness of the collection of relevant studies. Two investigators (H. M. and S. H.) reviewed the search results independently to select the studies based on the inclusion and exclusion criteria described below and assessed the eligibility of each study. The full text of articles was retrieved for eligibility assessment and further analyses after the initial screening with title and abstract. Discrepancies in the literature review were resolved by discussion and consensus.

Studies were included if they were published in a peer-reviewed journal and described neurologic adverse events in adult patients attributable to ertapenem specifically and reported relevant clinical data. The exclusion criteria included the following: (1) the full text not published in English; (2) review articles; and (3) conference abstracts.

### Data Extraction

We extracted demographic information and clinical data from each eligible study, including the following: indication for ertapenem use, patient comorbidities, signs and symptoms of ertapenem neurotoxicity, electroencephalographic (EEG) findings if performed, presence or absence of CNS conditions, presence or absence of renal dysfunction, dosing of ertapenem, time to onset of a neurological adverse event, outcomes, and the Naranjo Adverse Drug Reaction Probability Scale [9]. Renal dysfunction was defined as having either acute kidney injury or chronic kidney disease documented in the studies.

To supplement the systematic literature review, we also analyzed internal potential cases of ertapenem neurotoxicity at the UW Medicine health system between May 2021 and June 2023. A system-wide electronic health record query was performed to screen patients who developed neurological signs and symptoms after initiation of ertapenem during the same hospital encounter. Our electronic health record database was queried for the number of courses of ertapenem (≥2 doses) during the same interval to calculate a lower limit of incidence. Patients were considered to have received appropriate dosing of ertapenem when the dosing of ertapenem was 1 gram once daily in those with creatinine clearance >30 mL/minute and 500 mg once daily in those with creatinine clearance ≤30 mL/minute or on hemodialysis. The dose adjustment was assessed over the duration of ertapenem therapy. We calculated the Naranjo Adverse Drug Reaction Probability Scale for the internal cases identified and excluded cases with a low probability of drug-induced neurotoxicity.

## RESULTS

**Figure 1.**
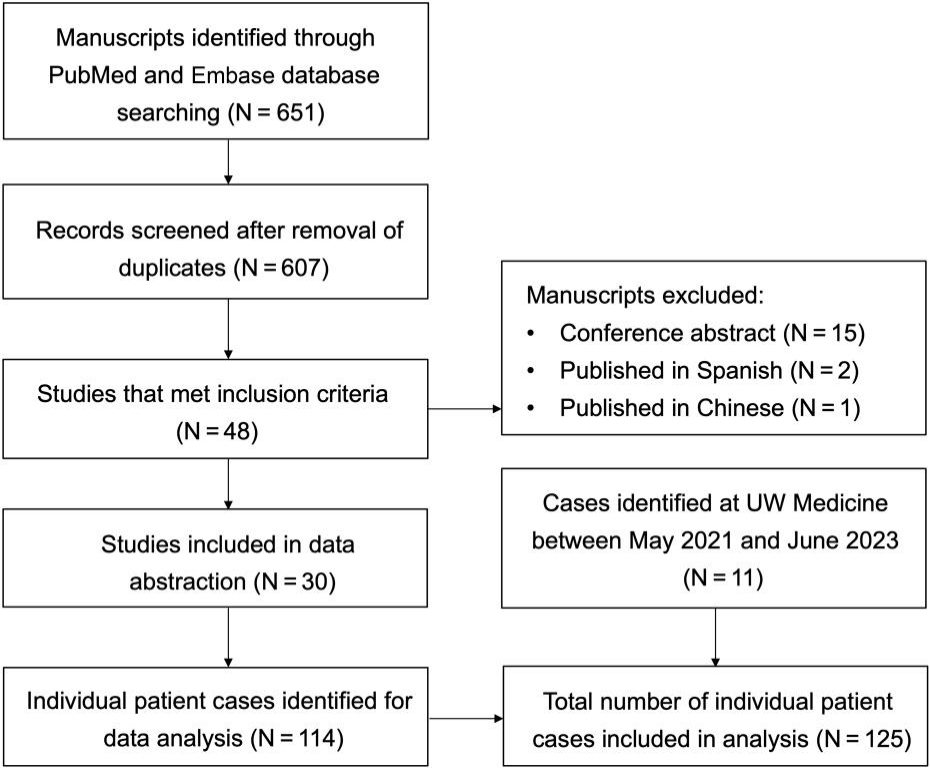
Preferred Reporting Items for Systematic Reviews and Meta-Analyses (PRISMA) flow diagram of study selection. Abbreviation: UW, University of Washington.

A total of 651 manuscripts were identified through the initial database search, and 30 studies met eligibility criteria for data extraction. Finally, a total of 125 individual patient cases were included in data analysis after adding 11 cases of suspected ertapenem neurotoxicity encountered at the UW Medicine health system (Figure 1). Characteristics of the included studies are summarized in Supplementary Appendix 1. Among 30 studies included, 18 were case reports, 8 case series, and 4 retrospective observational studies. Of 11 internal cases with suspected ertapenem neurotoxicity, 2 were classified as probable and 9 as possible.

Patient characteristics of suspected ertapenem neurotoxicity are summarized in Table 1. Average age of the patients was 72 years. Urinary tract infection was the most common indication for ertapenem use, which accounted for 45% of cases that documented the site of infection (29 of 64). While the majority of patients either had baseline chronic kidney disease and/or developed acute kidney injury during the encounter, only 15% (15/98) received inappropriately high ertapenem dosing based on kidney function. Preexisting CNS conditions or a history of seizure disorder were present in 42% of cases that documented baseline comorbidities. Of those that could be assessed using the Naranjo Adverse Drug Reaction Probability Scale, cases were fairly evenly split between probable (55% [22/40]) and possible (45% [18/40]).

**Table 1.** Patient Characteristics of Suspected Ertapenem Neurotoxicity

Characteristic	N = 125
Age, y, mean ± SD	72 ± 11
Male sex	70/109 (64)
Renal dysfunction^a^	66/106 (62)
End-stage renal disease	24/106 (23)
Predisposing CNS condition or history of seizure disorder	28/67 (42)
Received inappropriately high dose of ertapenem	15/98 (15)
Time to onset after starting ertapenem, d, median (IQR)	4 (3–9)
EEG performed and documented	25/125 (20)
Treatment	
Discontinuation of ertapenem	65/66 (98)
Antiepileptic medications used	54/105 (51)
Outcome	
Complete resolution/back to baseline	50/59 (85)
Partial recovery	1/59 (2)
Deceased	8/59 (14)
Naranjo Adverse Drug Reaction Probability Scale	
Definite	0/125 (0)
Probable	22/125 (18)
Possible	18/125 (14)
Unable to be calculated	85 of 125 (68)

Data are presented as no./No. (%) unless otherwise indicated.

Abbreviations: CNS, central nervous system; EEG, electroencephalogram; IQR, interquartile range; SD, standard deviation.

^a^Renal dysfunction includes both acute kidney injury and chronic kidney disease.

**Figure 2.**
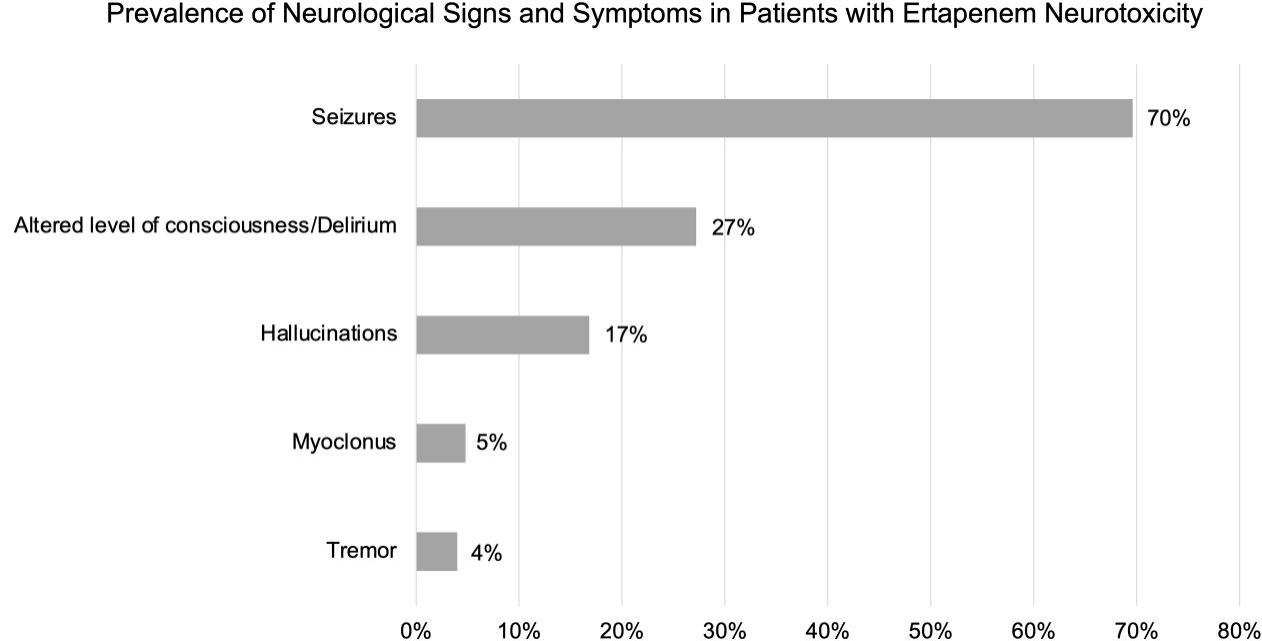
Clinical manifestations of ertapenem neurotoxicity. Clinical manifestations of ertapenem neurotoxicity were reported as a percentage of the total cohort (N = 125).

Patients developed neurological signs and symptoms attributable to ertapenem after a median of 4 days (interquartile range, 3–9 days). The most common clinical manifestation of suspected ertapenem neurotoxicity was seizures (70%), followed by altered level of consciousness/delirium (27%) and hallucinations (17%) (Figure 2). Myoclonus and tremor were seen in 5% and 4% of the cases, respectively. Less common clinical manifestations included gait abnormality/ataxia (n = 4), limb weakness (n = 4), asterixis (n = 3), nonconvulsive status epilepticus (n = 2), nystagmus (n = 2), numbness (n = 2), dysarthria (n = 2), suicidal ideations (n = 1), dysphagia (n = 1), and rigidity and clonus (n = 1). Among 25 patients with an EEG performed and documented, 13 cases were characterized by generalized or diffuse slow waves, and electrographic seizure activities were confirmed in only 4 cases.

After development of neurotoxicity, ertapenem was discontinued in all patients but one. Antiepileptic medications were used in 51% of cases (54/105), although this number is unreliable due to a lack of standardized reporting format.

The vast majority of patients had complete resolution of neurologic symptoms (85% [50/59]). Eight patients died, but only 1 death was attributed to seizures. Time to recovery after ertapenem discontinuation was not well documented in the literature.

Finally, between May 2021 and June 2023, 11 possible or probable cases were identified at UW Medicine out of 1121 courses of ertapenem administered during that time. This yields an incidence of 1 in 102 courses.

## DISCUSSION

This systematic review summarizes the current evidence and clinical characteristics of ertapenem neurotoxicity based on 30 studies. The existing literature consists mostly of case reports and case series, highlighting the need for further investigations to more accurately evaluate the true incidence, the associations with proposed risk factors, and the clinical manifestations of ertapenem neurotoxicity. For comparison, 3 systematic reviews on cefepime neurotoxicity performed their data analyses based on 71, 37, and 92 articles, respectively [10–12]. The relative paucity of studies on ertapenem neurotoxicity compared to cefepime neurotoxicity does not necessarily mean that it is comparatively rare. Ertapenem neurotoxicity may potentially be underdiagnosed and understudied, as suggested by the recent pharmacovigilance study using the FDA postmarketing drug safety surveillance system, which reported the highest delirium rate with ertapenem.

Based on our findings, typical patients with ertapenem neurotoxicity were older adults with renal dysfunction and/or a preexisting CNS condition (eg, a history of stroke, brain tumor, traumatic brain injury, or epileptic seizures). Risk factors for developing β-lactam neurotoxicity have been evaluated for other β-lactams (particularly penicillin, cefepime, and imipenem) and have been identified as higher dose, renal dysfunction, brain lesions, or known epilepsy [13]. Although it is reasonable to assume that ertapenem neurotoxicity is also associated with those risk factors, the unique pharmacokinetics of ertapenem may set it apart from the other β-lactam antibiotics, such as its long half-life, highly protein-bound nature, and unknown cerebrospinal fluid (CSF) penetration. In the presence of hypoalbuminemia, higher unbound ertapenem concentrations may lead to higher serum and possibly CSF concentrations [14, 15]. The paucity of available data on serum albumin levels in the case reports and case series included in our systematic review made it difficult to evaluate the prevalence of hypoalbuminemia among patients with ertapenem neurotoxicity.

The true independent predictors for developing ertapenem neurotoxicity remain to be elucidated. Few studies have been performed to investigate the risk factors of ertapenem neurotoxicity, and the results were not consistent. For example, a matched case-control study by Lee and colleagues found that the independent predictors associated with ertapenem-associated seizures were prior stroke, undergoing brain imaging within 1 year, anemia, and thrombocytopenia, but not older age and renal dysfunction [16]. On the other hand, a retrospective cohort study by El Nekidy and colleagues examining seizures associated with ertapenem use in patients undergoing hemodialysis for end-stage renal disease suggested that the currently approved ertapenem dose of 500 mg daily may impose a risk of developing neurotoxicity in these patients [17]. Further validation by larger prospective cohort studies is warranted to elucidate independent predictors for developing ertapenem neurotoxicity.

Patients developed neurological signs and symptoms after a median of 4 days with seizures being the most common clinical manifestation. Despite approximately 70% of the patients presenting with seizures, generalized or diffuse slow waves were the most common EEG finding and few patients were confirmed to have epileptiform discharges. Potential explanations for this discrepancy include limited sensitivity of routine EEG for detecting epileptic seizure activity, the time lag between seizure event and EEG, the influence of sedative and antiepileptic medications, or EEG testing possibly being performed more often in patients with encephalopathy-type symptoms. Seizures being the most commonly reported symptom may be a notable clinical feature of ertapenem neurotoxicity compared to other β-lactam antibiotics. For example, a systematic review on cefepime neurotoxicity reported diminished level of consciousness being the most common clinical manifestation (80%), followed by disorientation/agitation (47%) and myoclonus (40%), with seizures being present only in 11% of patients. While ertapenem may have a seizure-inducing propensity possibly shared with other carbapenems [13, 18], reporting bias is a major concern. Many studies included in our systematic review examined only patients who developed seizures. Although 8 patients died out of 59 cases for which outcomes were described in detail, the mortality was attributed primarily to their underlying conditions and critical illness.

We report an incidence at our institution of 1 in 102 courses of ertapenem. This is a rough estimate of a lower limit of incidence because active surveillance for ertapenem neurotoxicity has not been performed here. The incidence of ertapenem neurotoxicity at UW Medicine was much higher than that of cefepime neurotoxicity calculated at the same institution (1 in 480 courses) [10], which is consistent with the aforementioned pharmacovigilance study that revealed ertapenem having the highest delirium reporting association [7].

This study suggests potential implications for monitoring after the initiation of ertapenem. Ertapenem is a parenteral carbapenem that is frequently used as a definitive therapy for ESBL-producing Enterobacterales for up to several weeks in OPAT or during complicated hospital stays. Although ertapenem is generally well tolerated even when continued for weeks in an OPAT setting [4, 5, 19], based on the results of our study, patients should be carefully monitored for neurological symptoms, particularly for the first 7–10 days after starting ertapenem, especially in older adults with renal dysfunction and/or preexisting CNS conditions. The importance of routine renal function monitoring and adjustment of ertapenem dosing as needed while on ertapenem therapy should also be emphasized, since inappropriately higher dose relative to renal function is a generally well-accepted risk factor for the development of β-lactam neurotoxicity [6, 17]. We agree with the Infectious Diseases Society of America's OPAT guidelines, which state that serial laboratory testing should be monitored in patients receiving OPAT [20]. As a result of our internal quality improvement work, we are now developing an educational outreach plan. The intention is to increase awareness of this possible toxicity among our prescribers.

Our study has several limitations. First, the vast majority of the articles included in the analysis were case reports or case series, which limits the conclusions we may draw. We acknowledge reporting bias as an important possibility. Because these reports extrapolate clinical manifestations and risk factors of other β-lactam neurotoxicities, symptoms or risk factors specific to ertapenem neurotoxicity could be underreported. In addition, a certain characteristic (eg, seizures) of ertapenem neurotoxicity may have been overrepresented compared to others due to difference in information availability, as discussed above. Finally, lack of standardized reporting makes it difficult to estimate the prevalence of patient characteristics and clinical manifestations. Despite these limitations, we believe that this systematic review provides an important basis for future studies and helps clinicians more carefully monitor for ertapenem neurotoxicity.

## CONCLUSIONS

Ertapenem neurotoxicity may be associated with renal dysfunction and predisposing CNS conditions and should be considered in patients who develop seizures, altered mental status, or hallucinations, especially within several days after the initiation of ertapenem. This systematic review underscores the need for raising awareness among clinicians and a large prospective study to assess the true incidence and outcomes of ertapenem neurotoxicity.

## Supplementary Data

Supplementary materials are available at *Open Forum Infectious Diseases* online. Consisting of data provided by the authors to benefit the reader, the posted materials are not copyedited and are the sole responsibility of the authors, so questions or comments should be addressed to the corresponding author.

## Supplementary Material

ofae214_Supplementary_Data

## Notes

***Author contributions.*** All authors have seen and approved the manuscript and contributed significantly to this work.

***Patient consent.*** The institutional review board of the University of Washington approved the study and waived written informed consent.
