# Supplementary material for: Characterizing Ertapenem Neurotoxicity: A Systematic Review and Experience at a Tertiary Medical Center
Source: Open Forum Infect Dis. 2024 Apr 16;11(5):ofae214. doi: 10.1093/ofid/ofae214 (PMC11075787; doi:10.1093/ofid/ofae214)
Supplement: ofae214_Supplementary_Data [file ofae214_supplementary_data.docx]

**APPENDIX 1: SYSTEMATIC REVIEW CITATIONS**

| Authors | Publication Year | Journal | Title | Study Design | Number of cases |
| --- | --- | --- | --- | --- | --- |
| Seto AH, et al. | 2005 | Ann Pharmacother | Ertapenem-associated seizures in a peritoneal dialysis patient | Case report | 1 |
| Saidel-Odes L, et al. | 2006 | Clin Infect Dis | History of cerebrovascular events: a relative contraindication to ertapenem treatment | Case series | 2 |
| Fica AE, Abusada NJ. | 2008 | Scand J Infect Dis | Seizures associated with ertapenem use in patients with CNS disorders and renal insufficiency | Case series | 3 |
| Ong C, et al. | 2008 | Int J Antimicrob Agents | Seizures associated with ertapenem | Case series | 2 |
| Neo HY, et al. | 2013 | Journal of Clinical Gerontology and Geriatrics | Higher than expected rates of seizures associated with the use of ertapenem in older hospitalized patients | Retrospective | 6 |
| Shea YF, et al. | 2013 | Int J Clin Pharm | Delayed recovery from ertapenem induced encephalopathy: case-report and a possible mechanism | Case report | 1 |
| Wen MJ, et al. | 2013 | Clin Nephrol | Acute prolonged neurotoxicity associated with recommended doses of ertapenem in 2 patients with advanced renal failure | Case series | 2 |
| Oo Y, et al. | 2014 | Intern Med J | Ertapenem-associated psychosis and encephalopathy | Case report | 2 |
| Soštaric N, et al. | 2014 | Int J Clin Pharmacol Ther | Ertapenem-associated seizures in a patient without prior CNS disorder or severe renal dysfunction | Case report | 1 |
| Apodaca K, et al. | 2015 | Psychosomatics | Ertapenem-Induced Delirium: A Case Report and Literature Review | Case report | 1 |
| Kara E, et al. | 2015 | Clin Nephrol | Ertapenem-induced acute reversible peripheral neuropathy in chronic kidney disease: 3 case reports | Case series | 3 |
| Lee KH, et al. | 2015 | J Clin Pharm Ther | The recommended dose of ertapenem poses a potential risk for central nervous system toxicity in haemodialysis patients - case reports and literature reviews | Case series | 4 |
| Lin H, Chew ST. | 2015 | Drug Saf Case Rep | Status Epilepticus and Delirium Associated with Ertapenem in a Very Elderly Patient with Chronic Kidney Disease and Silent Ischaemic Cerebrovascular Disease | Case report | 1 |
| Veillette JJ, Van Epps P. | 2016 | Consult Pharm | Ertapenem-Induced Hallucinations and Delirium in an Elderly Patient | Case report | 1 |
| Yılmaz F, et al. | 2016 | Ther Apher Dial | Ertapenem Associated With Seizures in Treatment of Pyelonephritis in a Chronic Peritoneal Dialysis Patient | Case report | 1 |
| Aydın A, et al. | 2017 | Consult Pharm | Seizure Induced by Ertapenem in an Elderly Patient with Dementia | Case report | 1 |
| Lee YC, et al. | 2017 | PLoS One | Risk factors associated with the development of seizures among adult patients treated with ertapenem: A matched case-control study | Retrospective | 33 |
| Sutton SS, et al. | 2017 | J Investig Med High Impact Case Rep | Ertapenem-Induced Encephalopathy in a Patient With Normal Renal Function | Case report | 1 |
| Hanna RM, et al. | 2018 | Antibiotics (Basel) | A Case of Ertapenem Neurotoxicity Resulting in Vocal Tremor and Altered Mentation in a Dialysis Dependent Liver Transplant Patient | Case report | 1 |
| Patel UC, Fowler MA. | 2018 | J Spinal Cord Med | Ertapenem-associated neurotoxicity in the spinal cord injury (SCI) population: A case series | Case series | 4 |
| Tahseen A, et al. | 2019 | J Clin Psychopharmacol | Atypical Presentation of Ertapenum-Associated Hallucinations in a Patient With Psychiatric Disease | Case report | 1 |
| Adams R, et al. | 2020 | BMJ Case Rep | Ertapenem-induced encephalopathy | Case report | 1 |
| Neo HY, et al. | 2020 | Intern Med J | Higher rates of carbapenem-related seizures in older hospitalised adults | Retrospective | 16 |
| Danés I, et al. | 2021 | Br J Clin Pharmacol | A case series of confusional states and other neurotoxic effects caused by ertapenem | Case series | 10 |
| El Nekidy WS, et al. | 2021 | Ann Pharmacother | Ertapenem Neurotoxicity in Hemodialysis Patients-Safe and Effective Dosing Is Still Needed: A Retrospective Study and Literature Review | Retrospective | 10 |
| Farrugia F, Abela M. | 2021 | Malta Medical Journal | Ertapenem-induced delirium | Case report | 1 |
| Klimko CV, et al. | 2021 | J Pharm Pract | Probable Encephalopathy and Spasticity in a Multiple Sclerosis Patient Following Carbapenem Administration: A Case Report and Brief Literature Review | Case report | 1 |
| Fernández-Rubio B, et al. | 2022 | Daru | Probable ertapenem-induced encephalopathy; case report and suggested alternatives for chronic prostatitis | Case report | 1 |
| Martínez Delgado S, et al. | 2022 | Rev Esp Enferm Dig | Ertapenem neurotoxicity in liver transplantation | Case report | 1 |
| Shahar S, et al. | 2022 | BMC Nephrol | Ertapenem-induced neurotoxicity in an end-stage renal disease patient on intermittent haemodialysis: a case report | Case report | 1 |
